# A Case of Faith Cure

**Published:** 1886-03

**Authors:** 


### A CASE OF FAITH CURE.

*Editors Atlanta Medical and Surgical Journal:*

Your fair and candid treatment of the so-called "Faith Cure" question, in December JOURNAL, has induced me to send you copies of "Valley of Baca," and "From Baca to Beulah," by Miss Jennie Smith, the lady who never walked for sixteen years, but, like the cripple spoken of in Acts III., was carried to the temple. Since her healing she has been an effective, traveling evangelist in the railroad department of the W. C. T. U.

No one who hears her preach will doubt her honesty and sincerity, we think.

There has probably been no more remarkable faith cure in the nineteenth century, and its complete history, as given by herself, with the testimony of her physician, is well worthy a thoughtful perusal by the progressive practitioner.

Very truly, B.

Baltimore, Md., February 1, 1886.

